# 4-O-Methylascochlorin-Mediated BNIP-3 Expression Controls the Balance of Apoptosis and Autophagy in Cervical Carcinoma Cells

**DOI:** 10.3390/ijms232315138

**Published:** 2022-12-01

**Authors:** Yuna Cho, Yun-Jeong Jeong, Kwon-Ho Song, Il-Kyung Chung, Junji Magae, Taeg Kyu Kwon, Yung-Hyun Choi, Jong-Young Kwak, Young-Chae Chang

**Affiliations:** 1 Research Institute of Biomedical Engineering and Department of Cell Biology, School of Medicine, Catholic University of Daegu, Daegu 42472, Republic of Korea; 2 Department of Biotechnology, Catholic University of Daegu, Gyeongsan-Si 38430, Republic of Korea; 3 Magae Bioscience Institute, 49-4 Fujimidai, Tsukuba 300-1263, Japan; 4 Department of Immunology, School of Medicine, Keimyung University, Daegu 42601, Republic of Korea; 5 Department of Biochemistry, Dong-eui University College of Korean Medicine, Busan 47227, Republic of Korea; 6 Department of Pharmacology, School of Medicine, Ajou University, Suwon 16499, Republic of Korea

**Keywords:** 4-O-methylascochlorin, BNIP-3, HIF-1α, autophagy, apoptosis, anticancer activity

## Abstract

4-O-methylascochlorin (MAC) is a 4-fourth carbon-substituted derivative of ascochlorin, a compound extracted from a phytopathogenic fungus *Ascochyta viciae*. MAC induces apoptosis and autophagy in various cancer cells, but the effects of MAC on apoptosis and autophagy in cervical cancer cells, as well as how the interaction between apoptosis and autophagy mediates the cellular anticancer effects are not known. Here, we investigated that MAC induced apoptotic cell death of cervical cancer cells without regulating the cell cycle and promoted autophagy by inhibiting the phosphorylation of serine-threonine kinase B (Akt), mammalian target of rapamycin (mTOR), and 70-kDa ribosomal protein S6 kinase (p70S6K). Additional investigations suggested that Bcl-2/adenovirus E1B 19 kDa protein-interacting protein 3 (BNIP-3), but not Hypoxia-inducible factor 1 alpha (HIF-1α), is a key regulator of MAC-induced apoptosis and autophagy. BNIP-3 siRNA suppressed MAC-induced increases in cleaved- poly (ADP-ribose) polymerase (PARP) and LC3II expression. The pan-caspase inhibitor Z-VAD-FMK suppressed MAC-induced cell death and enhanced MAC-induced autophagy. The autophagy inhibitor chloroquine (CQ) enhanced MAC-mediated cell death by increasing BNIP-3 expression. These results indicate that MAC induces apoptosis to promote cell death and stimulates autophagy to promote cell survival by increasing BNIP-3 expression. This study also showed that co-treatment of cells with MAC and CQ further enhanced the death of cervical cancer cells.

## 1. Introduction

Human cervical cancer is a malignant tumor that affects about 500,000 women globally each year, with half of the cases leading to death [1,2]. Most cervical cancer patients have limited symptoms at the early stages of the disease, meaning they are diagnosed with advanced-stage disease [3,4]. Particularly, cervical cancer patients with advanced-stage disease and recurrence have a low 1 year relative survival rate of, about 10–20% [5]. The current primary treatments for cervical cancer are surgery, chemotherapy, and radiation therapy, but these are ineffective in advanced metastasis [6,7]. Thus, additional studies are needed to identify new therapeutic agents and improved treatment strategies that induce cell death in cervical cancer cells.

Apoptosis and autophagy are major forms of programmed cell death and determine the fate of cancer cells by balancing cell survival and death [8]. Apoptosis is distinguished by certain morphological features such as cell shrinkage and DNA fragmentation, leading to cell death [9]. The loss of the ability to control apoptosis is a characteristic of cancer cells, and results in indiscriminate growth of cells [10]. Autophagy is regulated by the activation of autophagic-related genes (ATG) and leads to the formation of autophagosomes, which promote the breakdown of damaged organelles, allowing cells to survive to starvation and other stresses. However, hyperactivity of autophagy also induces cell death, and this ‘autophagic cell death’ is associated with the elimination of cancer cells [11]. Autophagy can cooperate with apoptosis to induce cell death, or can act as positive and negative feedback on apoptotic cell death [12]. Thus, the apoptosis and autophagy pathway is an important target pathway for cancer chemotherapeutics [13,14,15].

Bcl-2/adenovirus E1B 19 kDa protein-interacting protein 3 (BNIP-3) is a pro-apoptotic protein with a BH3 domain that is localized to the mitochondrial membrane and causes apoptosis and autophagy. BNIP-3 causes the release of mitochondrial cytochrome c in a Bax/Bak-dependent way, culminating in the activation of caspase and apoptosis [16]. There is one evidence that BNIP-3 also binds competitively to Bcl-2, and so inhibits the binding of Bcl-2 and Beclin1, resulting in the enhancement of beclin1-mediated autophagy [17]. In addition, BNIP-3 interacts with the LC3 protein on the autophagosome, which contribute to mitochondrial autophagy [16]. Therefore, the regulation of BNIP-3 has an important role in the activation of apoptosis and autophagy.

4-O-methylascochlorin (MAC) is an ascochlorin derivative extracted from the phytopathogenic fungus *Ascochyta viciae* that has anti-cancer, and immunomodulatory properties, and positive effects on insulin resistance [18,19,20]. A previous study showed that MAC induces apoptosis mediated by the activation of caspase and degradation of poly (ADP-ribose) polymerase (PARP) in leukemia cells [21]. By inhibiting c-Myc expression, MAC has apoptotic effects of K562 leukemia cells without affecting normal blood cells by the stimulation of AMP-activated protein kinase (AMPK) and inhibition of mammalian target of rapamycin (mTOR) phosphorylation [20]. Furthermore, MAC induces autophagy by increased AMPK/Hypoxia-inducible factor 1 alpha (HIF-1α) expression in lung cancer cells [22]. However, the anticancer effect on MAC in solid cancer and the relationship between MAC-induced apoptosis and autophagy are currently unexplained.

In this study, we provide the first demonstration that MAC has cytotoxicity at the lowest concentration in cervical cancer cells among various solid cancer cells. Accordingly, we focused on the relationship between MAC-induced apoptosis and autophagy and underlying mechanisms of action in cervical cancer. We found that increased BNIP-3 expression is a major trigger of MAC-induced apoptosis and autophagy. Furthermore, MAC-induced autophagy facilitated cell survival, implying that MAC-induced apoptosis is a major mechanism of cell death.

## 2. Results

### 2.1. MAC Is Cytotoxic and Inhibits the Proliferation of Cervical Carcinoma Cells

**Figure 1:**
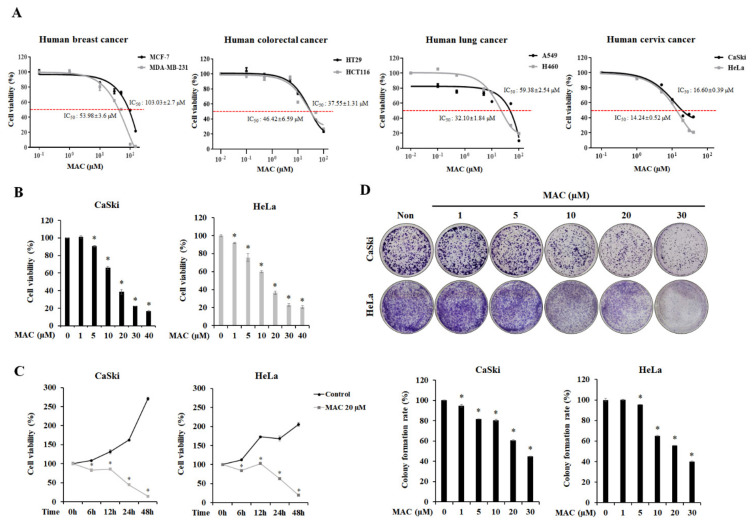
4-O-methylascochlorin (MAC) suppresses the cell viability and proliferation in cervical carcinoma cells. (**A**) Various cancer cells treated with different doses of MAC were evaluated using the WST-8 assay, and IC_50_ values were calculated using Excel. MAC IC_50_ values against each cell line are the mean of three separate investigations (*n* = 3). (**B**) CaSki and HeLa cells were treated with MAC at concentration of 1, 5, 10, 20, 30 and 40 μM for 24 h. (**C**) Cells were incubated with 20 μM of MAC for 6, 12, 24, and 48 h. Additionally, viability was determined by WST-8 assay. (**D**) Cells were incubated with indicated dose of MAC for 24 h, followed by staining with crystal violet to detect the cell proliferation. The rate of colony formation was measured by the absorbance at 562 nm. Values represent the means ± SD of triplicate assays. * *p* < 0.05 vs. control. Results were analyzed using one-way ANOVA followed by Bonferroni post hoc test.

To establish the cytotoxic effects of MAC on cancer cells, we first measured cell viability in various cancer cell lines including breast, colorectal, lung, and cervical cancer (Figure 1A). MAC had low potency in lung, colorectal, and breast cancer cell lines, with half-maximal-inhibitory concentrations values (IC_50_ values) > 30 μM, but had higher potency in CaSki and HeLa cervical carcinoma cells, with IC_50_ values of 16.6 μM and 14.24 μM, respectively. Therefore, the anti-cancer effect of MAC was most pronounced in cervical carcinoma cells. At 24 h after treatment, MAC significantly tended to reduce the viability of CaSki at concentrations less than 5 μM and HeLa at concentrations less than 1 μM (Figure 1B). At 20 μM, MAC decreased the viability of CaSki and HeLa cells by approximately 3-fold and 2-fold, respectively (Figure 1C). To determine the inhibitory effects of MAC on cell proliferation, a colony formation assay was carried out. MAC concentration-dependently reduced the number of cell colonies (Figure 1D). These results suggest that MAC inhibits cell viability and proliferation in cervical carcinoma cells.

### 2.2. MAC Induces Apoptosis of Cervical Carcinoma Cells

**Figure 2:**
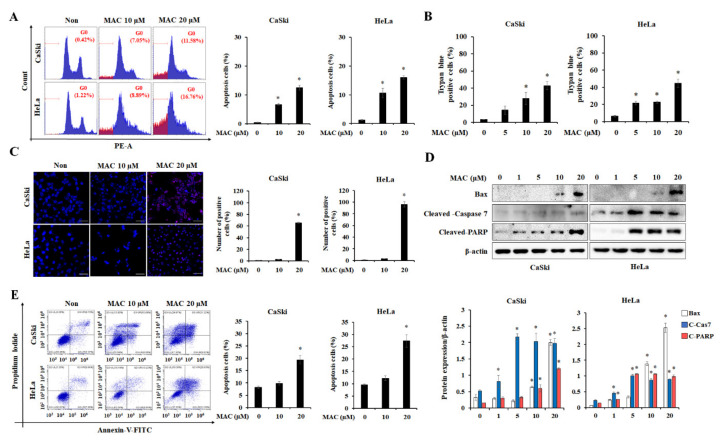
MAC triggers cell-death by inducing apoptosis in cervical carcinoma cells. (**A**) Cells were treated for 24 h with the indicated concentration of MAC, and cell cycle distribution was examined using flow cytometry. (**B**) Cells were stained by Trypan blue after being treated for 24 h with MAC at concentration of 5, 10 and 20 μM. The number of positive cells was counted by microscope. (**C**) Cells stained with Hoechst 33,342 (blue) as well as Propidium iodide (PI) (red) were observed using a fluorescent microscope with MAC of 10, and 20 μM for 24 h. The ImageJ program was used to calculate the percentage of PI positive cells. Scale bars were 50 µm. (**D**) CaSki and HeLa cells were treated with various concentration of MAC (0, 1, 5, 10, and 20 μM). The protein levels of Bax, cleaved caspase-7, cleaved-poly (ADP-ribose) polymerase (PARP) were determined by Western blot analysis. β-actin was used to control for loading. Relative expression density of each protein was measured by ImageJ program. (**E**) Cells were treated with indicated drug concentration (10 and 20 μM) for 24 h. The proportion of apoptotic cells were assessed using a flow cytometry after cells were labeled with Annexin-V-FITC and PI. Data represent the means ± SD of three independent experiments. * *p* < 0.05 vs. control. Results were analyzed using one-way ANOVA followed by Bonferroni post hoc test.

To determine whether the inhibitory effect of MAC on cell proliferation is related to cell-cycle arrest, flow cytometry was conducted by staining the cells with propidium iodide (PI) solution. MAC reduced the proportion of cells in the G2 phase and caused the accumulation of cells in the sub-G0 phase, without altering the proportion of cells in the G1 phase (Figure 2A), suggesting that MAC does not regulate cell-cycle arrest. MAC also increased the proportion of cells stained with trypan blue, a stain for dead cells, in a concentration-dependent manner (Figure 2B). The level of PI (red) stained cells, which represent dead cells, was increased by MAC (Figure 2C). These results collectively indicate that MAC induces cell death in cervical cancer cells. Western blotting was performed to investigate whether MAC induces the expression of apoptosis–related proteins. MAC at 20 μM significantly increased the expression of Bax, cleaved caspase-7, and cleaved-PARP in CaSki and HeLa cells (Figure 2D). The results of annexin V-FITC/PI dual staining showed that MAC increased the proportion of late apoptotic cells relative to the number of viable cells to apoptotic cells (Figure 2E). These results indicate that MAC triggers apoptosis of cervical carcinoma cells.

### 2.3. MAC Facilitates Autophagosome-Lysosome Fusion and Induces Autophagy in Cervical Carcinoma Cells

**Figure 3:**
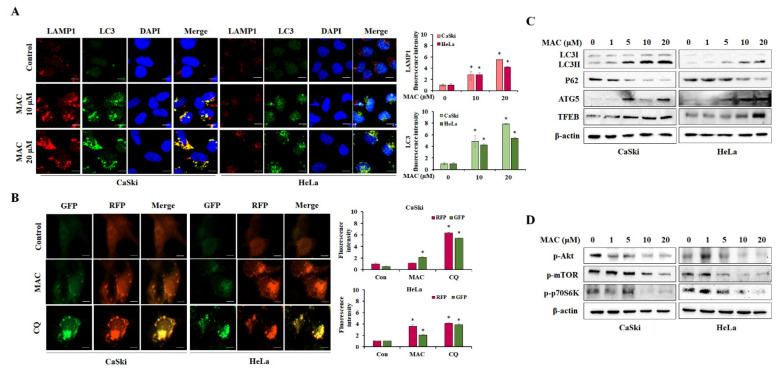
MAC induces autophagy in cervical carcinoma cells. (**A**) Cells were treated with MAC at concentration of 10 and 20 μM, respectively for 24 h and then subjected to immunofluorescence analysis using LC3 antibody (green) and Lysosomal associated membrane protein 1 (LAMP1) antibody (red). All nuclei were stained using DAPI (blue). The intensity of LC3 and LAMP1 puncta was measured by ImageJ program. Scale bars were 10 µm. (**B**) Plasmids encoding GFP-RFP-LC3 were transiently transfected into cells followed by treating with MAC (20 μM) and chloroquine (CQ) (50 μM) for 24 h, respectively. The intensity of GFP and RFP puncta was gauged by ImageJ program. Scale bars were 10 µm. (**C**) Cells were treated with various concentration of MAC (0, 1, 5, 10, and 20 μM) for 24 h. Western blot analysis was performed to examine LC3, p62, Autophagy-related gene 5 (ATG5), and transcription factor EB (TFEB) protein levels in each group and (**D**) verify the phosphorylation level of serine-threonine kinase B (Akt), mammalian target of rapamycin (mTOR), and 70-kDa ribosomal protein S6 kinase (p70S6K). Values represent the means ± SD of triplicate assays. * *p* < 0.05 vs. control. Results were analyzed using one-way ANOVA followed by Bonferroni post hoc test.

The major feature of autophagy is the formation of autophagosomes, which then fuse with lysosomes to induce the degradation of cargo by lysosomal hydrolases and permease [23]. LAMP1, a lysosomal membrane protein, has a major role in autophagosome-lysosome fusion. When the autophagosome and lysosome are fused, Lysosome associated membrane protein 1 (LAMP1) and LC3 are colocalized [24]. Therefore, to determine the effects of MAC on autophagy, we measured the immunofluorescence of LC3 and LAMP1. CaSki and HeLa cells were treated with 10 and 20 μM MAC for 24 h, which markedly increased in the formation of LAMP1 and LC3 puncta. In addition, a yellow overlay was observed in MAC-treated cells (Figure 3A), supporting the notion that MAC stimulates autophagy by inducing autophagosome-lysosome fusion. We analyzed the red and yellow puncta after transfection of cell with the GFP-RFP-LC3 plasmid to further determine whether MAC increases the accumulation of autophagosome. GFP-RFP-LC3 plasmid has been found to morphologically indicate autophagic flux. When cells are transfected with GFP-RFP-LC3, yellow puncta indicate autophagosome and red puncta indicate autolysosome [25]. GFP is sensitive to acidic PH in the lysosome and GFP fluorescence signals is quenched in the autolysosome [26,27,28].

As illustrated as Figure 3B, CQ led to an accumulation of yellow puncta which signals both RFP and GFP, indicating that CQ prevents the fusion of autophagosome and lysosome (positive cells). Yellow signals were boosted by MAC in comparison to control. These data collectively suggested that MAC induced autophagy by the accumulation of autophagosome in cervical carcinoma cells.

In addition, the results of the Western blot analysis showed that MAC significantly increased levels of LC3II/LC3I and ATG5, as well as increased levels of the transcription factor EB (TFEB), which regulates the autophagy-lysosomal pathway [29]. Next, we measured the expression of p62 (SQSTM1), which selectively binds to LC3 and undergoes autophagic degradation, in cervical carcinoma cells [30], while the level of p62 was reduced by MAC (Figure 3C). In addition, MAC concentration-dependently decreased the phosphorylation of Akt and mTOR, which form the major direct signaling pathway of autophagy. The phosphorylation of p70S6K, which signals downstream of mTOR, was also decreased by MAC (Figure 3D). These results suggest that MAC promotes autophagy by inhibiting the activity of the Akt/mTOR/p70S6K signaling pathway in cervical carcinoma cells.

### 2.4. BNIP-3 Mediates MAC-Induced Autophagy and Apoptosis in Cervical Carcinoma Cells

**Figure 4:**
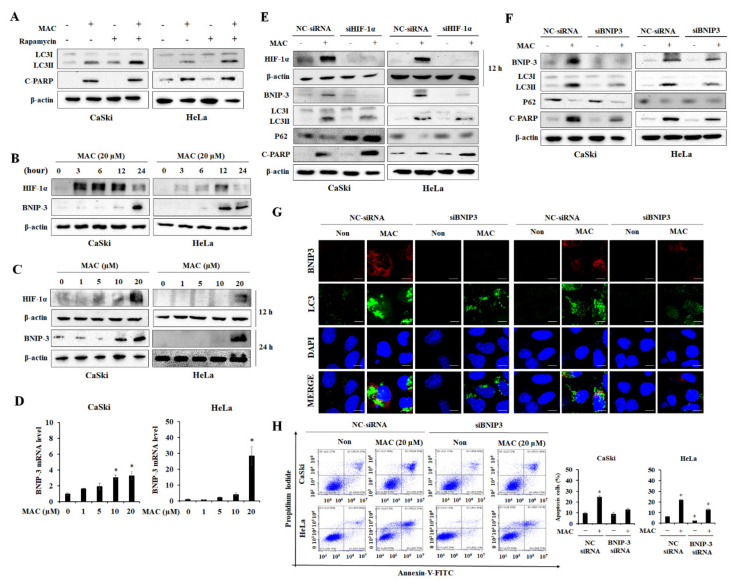
MAC-induced autophagy and apoptosis are regulated by Bcl-2/adenovirus E1B 19 kDa protein-interacting protein 3 (BNIP-3) expression rather than rapamycin and Hypoxia-inducible factor 1 alpha (HIF-1α) in cervical carcinoma cells. (**A**) Cells were treated with MAC (20 μM) for 24 h in the presence or absence of the rapamycin (1 μM), then protein levels of LC3 and cleaved-PARP were determined by Western blot analysis. (**B**) After the cells were treated with 20 μM of MAC, the protein levels of HIF-1α and BNIP-3 were checked for each indicated time (0, 3, 6, 12 and 24 h). (**C**) Cells were treated with various dose of MAC (0, 1, 5, 10 and 20 μM) for 12 h and 24 h, then Western blotting analyzed with antibodies against HIF-1α and BNIP-3, respectively. (**D**) Cells were incubated with concentration of MAC (0, 1, 5, 10 and 20 μM) for 24 h, then mRNA level of BNIP-3 was checked by RT-PCR analysis. (**E**) CaSki and HeLa cells were transfected with negative siRNA and HIF-1α siRNA then treated with MAC for 24 h. Subsequently, cell pellets were collected for Western blot to examine proteins levels of BNIP-3, LC3, p62 and cleaved-PARP. HIF-1α and its corresponding β-actin were confirmed after 12 h of treatment with MAC. (**F**) Cells were transfected with negative siRNA and BNIP-3 siRNA then treated with MAC for 24 h, the protein levels of BNIP-3, LC3, p62 and cleaved-PARP were examined by Western blot analysis. (**G**) After knockdown under the same conditions as (**F**), immunofluorescence analysis was performed using LC3-antibody (green) and BNIP-3 (red). All nuclei were stained using DAPI (blue). Scale bars were 10 µM. (**H**) The apoptotic cells were verified by PI/Annexin V-FITC staining. Values represent the mean ± SD of triplicate assays. * *p* < 0.05 vs. negative control. Results were analyzed using one-way ANOVA followed by Bonferroni post hoc test.

To establish the role of the Akt/mTOR/p70S6K pathway in MAC-induced autophagy and apoptosis, cells were co-treated with MAC and rapamycin, an inhibitor of mTOR. The increase in LC3II protein levels was significantly higher in cells treated with rapamycin and MAC than in cells treated with MAC alone, suggesting that the Akt/mTOR pathway is not the primary pathway involved in MAC-induced autophagy. In addition, the expression of cleaved-PARP did not vary substantially between cells treated with MAC and those treated with the combination of MAC and rapamycin (Figure 4A). These results indicate that the MAC-mediated inhibition of mTOR phosphorylation is not involved in MAC-induced apoptosis.

A previous study reported that MAC is a potent inducer of HIF-1α activity under normal O_2_ conditions [22]. BNIP-3 is a HIF-dependent gene product, and as such has a role in hypoxia-induced autophagy, which enables cancer cells to survive and enhance tumor progression [31]. Therefore, we measured the expression of HIF-1α and BNIP-3 in CaSki and HeLa cells following MAC treatment. As shown in Figure 4B, HIF-1α expression was increased by MAC in a time-dependent manner up to 12 h and then decreased at 24 h. However, BNIP-3 expression was increased at 24 h of MAC treatment. Based on these results, HIF-1α and BNIP-3 were evaluated at 12 h and 24 h of MAC treatment, respectively. MAC at 20 µM significantly increased HIF-1α and BNIP-3 expression (Figure 4C). HIF-1α is a transcription factor that regulates the mRNA level of BNIP-3. MAC increased the mRNA level of BNIP-3 (Figure 4D), which suggests that MAC increased BNIP-3 mRNA levels by stabilizing HIF-1α. Therefore, the role of HIF-1α in MAC-induced apoptosis and autophagy was investigated using HIF-1α siRNA transfected cells. HIF-1α siRNA prevented MAC-induced BNIP-3 and LC3II expression. The expression of p62 was reduced in the presence of MAC in cells transfected with negative control. However, HIF-1α did not affect the expression of p62 reduced by MAC and HIF-1α siRNA enhanced MAC-stimulated cleaved PARP expression (Figure 4E). These results suggest that HIF-1α is involved in MAC-induced autophagy but is not involved in MAC-induced apoptosis.

Next, to determine whether MAC-induced apoptosis was mediated by BNIP-3, CaSki and HeLa cells were transfected with siRNA targeting BNIP-3. In control cells, MAC induced BNIP-3 expression. In cells transfected with BNIP-3 siRNA, MAC did not alter the expression of BNIP-3. BNIP3 siRNA suppressed MAC-stimulated LC3II/LC3I expression and expression of cleaved-PARP. However, BNIP-3 siRNA did not affect the expression of p62 (Figure 4F). These results suggest that BNIP-3 is involved in MAC-mediated autophagy and apoptosis. Further, immunofluorescence was used to observe BNIP-3- and LC3-labeled vacuolation to confirm whether BNIP-3 mediates MAC-induced autophagy. BNIP-3 siRNA reduced the level of MAC-induced BNIP-3 red puncta and LC3 green puncta (Figure 4G). The results of flow cytometry showed that BNIP-3 siRNA inhibited the MAC-mediated increase in the proportion of apoptotic cells (Figure 4H). These results indicate that autophagy and apoptosis of cervical carcinoma cells by MAC are dependent on increased expression of BNIP-3 rather than increased HIF-1α expression.

### 2.5. Inhibition of Apoptosis Enhances MAC-Induced Autophagy in Cervical Carcinoma Cells

**Figure 5:**
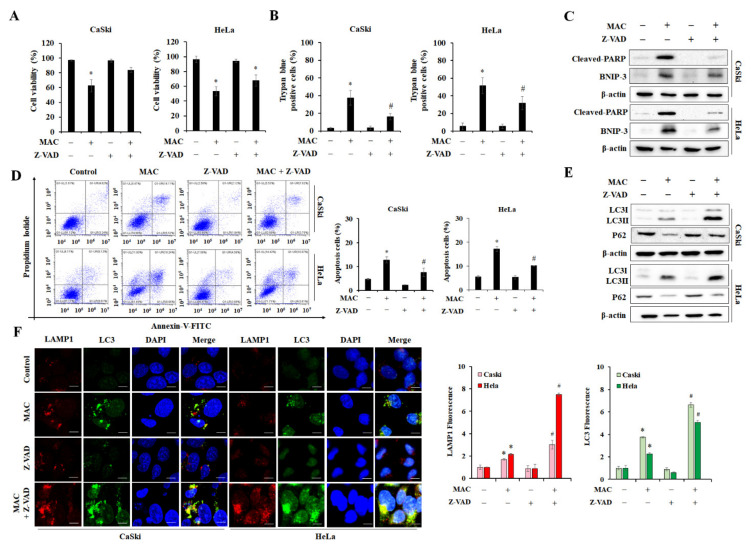
Inhibition of apoptosis enhances MAC-induced autophagy. (**A**,**B**) Cells were stained with trypan blue for detection of dead cells treated with MAC (20 μM) with or without Z-VAD-FMK (10 μM). (**C**) The protein levels of BNIP-3 and Cleaved-PARP were assessed with Western blotting analysis of CaSki and HeLa cells treated with or without MAC and Z-VAD-FMK, respectively. (**D**) Cells were labelled with annexin V-FITC/PI to detect the apoptotic cells. (**E**) The protein levels of LC3 and p62 were assessed with Western blotting analysis of CaSki and HeLa cells treated with or without MAC and Z-VAD-FMK, respectively. (**F**) Cells were treated with drugs for 24 h and then subjected to immunofluorescence analysis using LC3-antibody (green) and LAMP1 (red). All nuclei were stained using DAPI (blue). The intensity of LC3 and LAMP1 puncta was measured by ImageJ program. Scale bars were 10 µm. Data represent the mean ± SD of three independent experiments. * *p* < 0.05 vs. negative control and # *p* < 0.05 vs. MAC. Results were analyzed using one-way ANOVA followed by Bonferroni post hoc test.

We next investigated the relationship between the MAC-mediated reduction in cell viability and caspase-dependent apoptosis. The cell viability and the number of trypan blue-positive cervical carcinoma cells were analyzed in the presence of Z-VAD-FMK, a pan-caspase inhibitor. Z-VAD-FMK prevented the MAC-mediated decrease in cell viability and the MAC-mediated increase in the level of trypan blue-stained cells (Figure 5A,B). Z-VAD-FMK suppressed the induction of cleaved PARP by MAC. MAC-induced BNIP-3 expression tended to be decreased by Z-VAD-FMK (Figure 5C). In addition, Z-VAD-FMK prevented MAC from increasing the level of apoptotic cells (Figure 5D), indicating that MAC-stimulated cell death is due to caspase-dependent apoptosis. Next, the regulatory effect of apoptosis on MAC-induced autophagy was investigated. Treatment with Z-VAD-FMK alone did not alter the LC3II and p62 expression, whereas Z-VAD-FMK enhanced the MAC-induced increase in LC3II expression (Figure 5E). Immunofluorescence also showed that cotreatment with MAC and Z-VAD-FMK increased the formation of LC3 puncta and lysosomes compared with MAC treatment alone (Figure 5F). Therefore, activation of caspase pathway is crucial for MAC-stimulated apoptosis, Inhibition of caspase enhanced autophagy activity without the effect of MAC-induced BNIP-3 expression.

### 2.6. Inhibition of Autophagosome-Lysosome Fusion Enhances MAC-Induced Apoptosis in Cervical Carcinoma Cells

**Figure 6:**
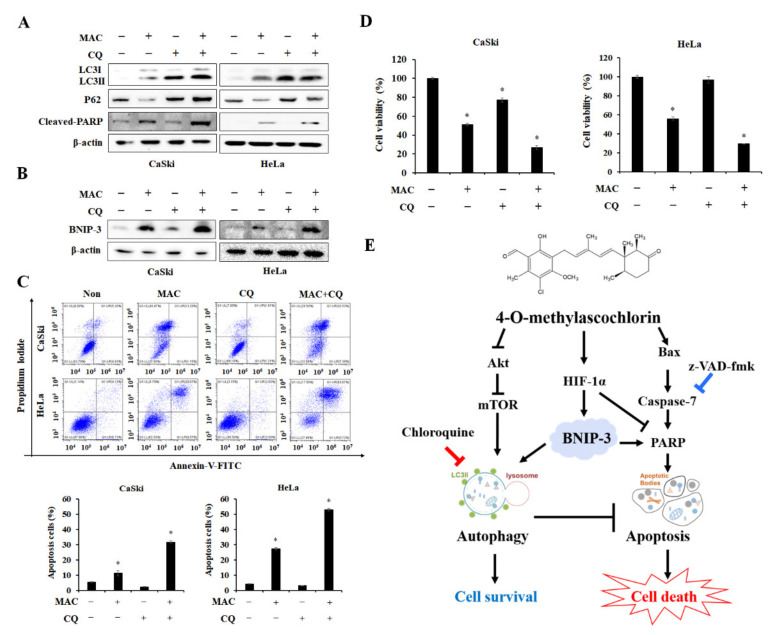
CQ enhances MAC-induced apoptosis in cervical carcinoma cells. (**A**) Cells were treated with MAC (20 μM) for 24 h in the presence or absence of CQ (50 μM), Western blotting analysis was used to determine the protein levels of LC3, P62 and cleaved-PARP in cell lysates. (**B**) After the treatment as (**A**) for 12 h, the protein level of BNIP-3 was conducted by Western blot analysis. (**C**) Cells were treated with MAC in the presence or absence of CQ, and double stained Annexin V-FITC/PI. The apoptotic cells were verified by Flow cytometry. (**D**) Cells were incubated with MAC and CQ for 24 h, then cell viability was conducted with WST-8 assay. Data represent the mean ± SD of three independent experiments. * *p* < 0.05 vs. control. Results were analyzed using one-way ANOVA followed by Bonferroni post hoc test. (**E**) Suggest molecular mechanism by which BNIP-3 expression regulates the balance of MAC-induced autophagy and apoptosis in cervical cancer cells.

The role of autophagy on MAC-stimulated apoptosis in cervical carcinoma cells was investigated using CQ, which inhibits autolysosome formation by blocking the fusion between the autophagosome and lysosome [32,33]. Treatment of cells with CQ alone stimulated LC3II and p62 accumulation without affecting the expression of cleaved-PARP. CQ blocked MAC-stimulated autophagy by inhibiting the p62 degradation, whereas CQ enhanced MAC-induced expression of cleaved-PARP (Figure 6A). Interestingly, cells treated with CQ and MAC had significantly higher expression of BNIP-3 compared to treatment of MAC alone (Figure 6B), suggesting that increased BNIP-3 expression mediated by cotreatment may stimulate apoptosis. Therefore, to confirm if CQ and MAC had synergetic effects on cell death, we measured apoptotic cell rate and cell viability. Cells co-treated with CQ and MAC had a higher ratio of late apoptotic cells than cells treated with MAC alone (Figure 6C). Similarly, MAC synergistically decreased cell viability (Figure 6D). Collectively, these results demonstrate that the CQ-mediated increase in LC3II, p62, and BNIP-3 expression promotes MAC-induced apoptosis and leads to cell death in cervical carcinoma cells. Therefore, the apoptotic effects of MAC were enhanced by blocking the fusion between autophagosomes and lysosomes.

## 3. Discussion

Ascochlorin, an isoprenoid antibiotic generated by the phytopathogenic fungus *Ascochyta viciae*, has anti-tumor activity in animal studies [19,34]. MAC, a methylated derivative of ascochlorin, also has potential anti-tumor effects [18,35]. MAC is known to induce apoptosis and autophagy in various cancer cell lines [20,21,22,36]. However, the role and major signaling pathway of MAC-stimulated autophagy on its anti-tumor activity were not known before the current study. In this study, we demonstrated the importance of BNIP-3 in the relationship between MAC-induced apoptosis and autophagy and proposed a strategy for inducing autophagy in cervical cancer cells using MAC that could have therapeutic implications. We analyzed the anticancer effect of MAC on cervical carcinoma cells by various methods. MAC induced cell cytotoxicity and disrupted of the cell cycle by increasing the sub-G0 cell population [20,37]. MAC induced apoptosis by increasing the protein levels of Bax, cleaved caspase-7, and PARP, suggesting that MAC has an anticancer effect on cervical carcinoma cells (Figure 2). In addition, MAC increased the protein levels of LC3, ATG5, and transcription factor EB, which are molecules required to initiate autophagy [38,39,40,41]. In contrast, MAC reduced protein levels of p62, which recognizes ubiquitinated proteins [42]. Through these results, we showed that MAC effectively increases apoptosis and autophagy in cervical carcinoma cells.

Autophagy is mediated by a multitude of signaling pathways. Some of them converge at mTOR, a cellular energy sensor that detects changes in the ATP/AMP ratio [39,43]. MAC reduced the phosphorylation of Akt, mTOR, and p70S6K (Figure 3). However, rapamycin increased MAC-induced increases in LC3II expression without affecting MAC-induced cleaved-PARP levels (Figure 4). These results suggest that MAC-induced autophagy was induced not only by the mTOR pathway but also by other pathways, and that MAC-induced apoptosis does not involve the mTOR pathway.

Previous studies have reported that MAC potently stabilizes the HIF-1α protein [18,44]. HIF-1α is an upstream regulator of BNIP-3 and contributes to cell survival by increasing BNIP-3 expression and mitochondrial autophagy [45]. As expected, MAC increased HIF-1α and BNIP-3 expression in cervical carcinoma cells. HIF-1α siRNA increased the MAC-induced increase in cleaved PARP expression while inhibiting the expression of autophagy markers and BNIP-3. These results indicate that the MAC-induced increase in HIF-1α expression prevents apoptosis in cervical carcinoma cells. These findings are similar to those showing the MAC stabilizes the HIF-1α protein to induce survival of breast cancer cells by stimulating epithelial-mesenchymal transition [46]. Interestingly, while BNIP-3 expression is regulated by HIF-1α, which is involved in cell survival, BNIP-3 is known to be a pro-apoptotic Bcl-2 family protein, which contributes to mitochondrial malfunction and cell death via apoptosis. BNIP-3 induces apoptosis by opening the mitochondrial permeability transition pore when the expression of Bax and Bak is increased [47,48,49]. In the current study, MAC increased the expression of Bax as well as BNIP-3, indicating that MAC may stimulate apoptosis by inducing the expression of BNIP-3. BNIP-3, as a critical effector protein in autophagy, also enhances hypoxia-induced autophagy by interacting with LC3 [50]. The MAC-induced increases in the expression of both LC3 and cleaved-PARP were reduced in BNIP-3-silenced cells (Figure 4). Therefore, the induction of BNIP-3 expression is essential for MAC-induced apoptosis and autophagy.

Autophagy is involved in cell survival to maintain intracellular homeostasis. Additionally, autophagy can facilitate cell death [9,51]. The inhibition of caspase activity further increased MAC-induced autophagy, resulting in the inhibition of cell death. These findings showed that MAC-induced autophagy plays a role in regulating intracellular homeostasis. Many recent studies have been conducted using CQ, which prevents autolysosome production, in combination with chemotherapy to investigate the role of CQ in autophagy inhibition. CQ promotes caspase-dependent apoptosis of primary effusion lymphoma cells by inhibiting autophagy [52]. Combined therapy with CQ and enzalutamide induced apoptosis and reduces tumor growth, thereby enhancing the anti-cancer effects of enzalutamide in bladder cancer [53]. As expected, CQ enhanced MAC-induced apoptosis of cervical carcinoma cells, and this effect was caused by the accumulation of LC3 and BNIP-3. Therefore, the CQ-mediated suppression of the MAC-induced autophagy represents an apoptotic strategy that ultimately results in cell death.

In conclusion, our study demonstrated that induced BNIP-3 expression by MAC directly promotes apoptosis and autophagy and lead to cell death in cervical carcinoma cells. This study provides that MAC can lead to autophagy through inhibition of mTOR pathway and upregulation of HIF-1α/BNIP-3 signaling pathway. However, our results showed the activation of autophagy by HIF-1α expression suppresses apoptotic cell death under MAC treatment. Together, our present data highlight the need to regulate autophagy activity for BNIP-3 overexpression-induced apoptotic cell death. Therefore, we suggest a combination treatment of MAC and CQ to overcome the limitations of MAC alone for cancer therapy.

## 4. Materials and Methods

### 4.1. Cell Culture and Materials

Human lung (A549, H460), colorectal (HT29, HCT116), breast cancer (MDA-MB-231, MCF7) and cervical (CaSki, HeLa) carcinoma cell lines were purchased from the American Type Culture Collection (ATCC) (Rockville, MD, USA). Cancer cells were cultured in Dulbecco’s Modified Eagle’s Medium (DMEM) medium and Roswell Park Memorial Institute (RPMI) 1640 medium (Hyclone; GE Healthcare Life Sciences, Logan, UT, USA) containing 10% fetal bovine serum (FBS) (Gibco, Grand Island, NY, USA) and 1% penicillin (Hyclone; GE Healthcare Life Sciences, Logan, UT, USA). MAC was provided by Chugai Pharmaceutical Company (Tokyo, Japan).

### 4.2. Cell Viability Assay

Cells were seeded at a density of 5 × 10^3^ cells/well in 96-well plates and incubated overnight in a humidified atmosphere of 5% CO_2_ at 37 °C. The cells were incubated with MAC at the indicated concentration for the indicated time, and then Quanti-Max reagents (Biomax, Seoul, Korea) were applied to the cells for 2 h. The absorbance of each well was then read at 450 nm using a microplate reader (VersaMax microplate reader; Molecular Devices, Sunnyvale, CA, USA).

### 4.3. Colony Formation Assay

Human cervical carcinoma cells were seeded and incubated in 6-well plates at a density of 5 × 10^3^ cells/well until they were approximately 80% confluent. The cells were treated with MAC (1, 5, 10, 20, and 30 μM) for 1 day, and then fixed with 100% methanol for 5 min and stained with 0.5% crystal violet for 30 min at room temperature. The cells were photographed after washing with phosphate-buffered saline (PBS) three times.

### 4.4. Cell Cycle Analysis

Human cervical carcinoma cells were seeded at a density of 4 × 10^5^ cells in 60 mm dishes and incubated overnight at 37 °C. After treatment with MAC for 24 h, the cells were harvested with trypsin EDTA (disodium ethylenediaminetetraacetic acid) (Hyclone; GE Healthcare Life Sciences), washed with PBS, and then fixed with 75% ethanol overnight at 4 °C. Cells were stained with propidium iodide (PI; 1 mg/mL) (Beckman Coulter, Miami, FL, USA) and RNase. The cell cycle was analyzed using flow cytometry (Beckman Coulter, Miami, FL, USA).

### 4.5. Trypan Blue Staining

Human cervical carcinoma cells were seeded at a density of 4 × 10^5^ cells in 60 mm dishes and incubated overnight at 37 °C. Subsequently, the media was changed to new RPMI media, and cells were treated with 10 and 20 μM MAC for 24 h. The cells were then stained with trypan blue (0.4%) (Sigma Aldrich, St. Louis, MO, USA), and dead cells were counted with a microscope (Nikon Eclipse TS100, Nikon Corporation, Tokyo, Japan).

### 4.6. Hoechst 33,342 and PI Double Staining

Human cervical carcinoma cells were seeded at a density of 4 × 10^5^ cells in 60 mm dishes and incubated overnight at 37 °C. Subsequently, the media was changed to new RPMI media, and cells were treated with 10 and 20 μM MAC for 24 h. The cells were then stained with Hoechst 33,342 (Immunochemistry Technologies LLC, Bloomington, MN, USA) and PI (Beckman Coulter) for 15 min at room temperature in the dark. Then, the cells were observed using a confocal microscope (Leica Microsystems, Wetzlar, Germany).

### 4.7. Flow Cytometry for Apoptosis

During the initial stage of apoptosis, Phosphatidylserine is exposed at cell surface, and Annexin V protein is used as a marker of apoptosis because it is bound for cells exposed to Phosphatidylserine [54]. Therefore, we used Annexin V-FITC/PI staining to approach study for apoptotic cells. Human cervical carcinoma cells were seeded at a density of 4 × 10^5^ cells in 60 mm dishes and incubated overnight at 37 °C. After treatment with the indicated drugs for 24 h, the cells were stained for apoptosis analysis using a FITC (Fluorescein isothiocyanate) annexin V kit (BD Biosciences, Franklin Lakes, NJ, USA). Cells were stained with FITC Annexin V, PI labeling solution for 15 min at room temperature according to manufacture protocol. Finally, stained cells were analyzed by flow cytometry (Beckman Coulter, Miami, FL, USA).

### 4.8. RT-PCR

Human cervical carcinoma cells were seeded at a density of 4 × 10^5^ cells in 60 mm dishes and incubated overnight at 37 °C, and then contain the fresh medium with the indicated concentration of MAC. After 24 h, total RNA was extracted using TRIzol reagent (Thermo Fisher Scientific, Waltham, NJ, USA), as directed by protocol. cDNA was synthesized from 1 µg of total RNA. After cDNA synthesis, RT-PCR was performed with SYBR green PCR master mix (Cosmo Genetech, Daejeon, Korea) and BNIP-3 primers (Bioneer Co., Daejeon, Korea): 5′-GTCATGAAGAAAGGGGGCAT-3′ and 5′-CTGGTGGAGGTTGTCAGACG-3′, using a real-time PCR instrument (Bio-Rad Laboratories, Hercules, CA, USA). β-actin was used as control.

### 4.9. Western Blot Analysis

Lysis buffer was used to produce cell lysates as previously described [22]. The collected protein lysates were separated by electrophoresis on an SDS-PAGE gel and transferred onto Immobilon-P (Merck Millipore, Billerica, MA, USA) or nitrocellulose membranes (Merck Millipore, Billerica, MA, USA) using a semi-dry system (Bio-Rad, Seoul, Korea). Primary antibodies for Bax (sc-7480), P62 (sc-28359), BNIP-3 (sc-56167), p-mTOR (sc-101738), p-p70S6K (sc-7948-R), β-actin (sc-47738) were purchased from Santa Cruz (Dallas, TX, USA), and primary antibodies for p-Akt (#9271), cleaved caspase-7 (#8438), cleaved PARP (#5625), ATG5 (#12994), and LC3 (#4108) were purchased from Cell Signaling Technology (Danvers, MA, USA). Following primary antibody incubation, the membrane was incubated at room temperature with secondary antibodies (Thermo Fisher Scientific), namely goat anti-mouse immunoglobulin G (IgG; #31430) or goat anti-rabbit IgG (#65-6120) diluted in 3% skim milk. The signals were visualized using chemiluminescence (GE Healthcare, Chicago, IL, USA), then detected by ChemiDOC XRS (Bio-Rad Laboratories) and Davinch-Chemi^TM^ Imaging system (Davinch-K, Seoul, Korea)

### 4.10. Immunofluorescence

Human cervical carcinoma cells were seeded on glass slides (SPL Life Sciences, Pocheon-si, Korea) at 50% confluence and incubated until 80% influence was achieved. After treatment with drugs for 24 h, cells were fixed using 100% methanol and then permeabilized with 0.2% Triton X-100. Cells were blocked with 10% normal goat-serum (#31872, Invitrogen; Thermo Fisher Scientific, Waltham, MA, USA) in PBS. Cells were incubated with LAMP1, LC3, and BNIP-3 antibodies overnight at 4 °C. Cells were treated with Alexa-594-conjugated anti-mouse IgG and Alexa-488-conjugated goat anti-rabbit IgG antibodies (1:500). Cells were washed with 0.05% Tween-20 in PBS. Then, cells were stained with DAPI (Sigma Aldrich) for 4 min to detect the nucleus. Fluorescent signals were visualized using a confocal microscope (Leica Microsystems, Wetzlar, Germany).

### 4.11. GFP-RFP-LC3 Plasmid Transfection and Autophagy Flux

Human cervical carcinoma cells were seeded at 50% confluence on glass slides and incubated at 37 °C. When the density of cells reached 70–80%, the GFP-RFP-LC3 plasmid (Addgene, Watertown, MA, USA) was transfected using lipofectamine 2000 (Invitrogen; Thermo Fisher Scientific, lnc.) in accordance with the instructions of transfection reagent. New RPMI media containing 0.5% FBS was replaced after transfected for dd4 h. Subsequently, Cells were treated with indicated concentration of MAC and CQ. After treatment with these drugs for 24 h, cells were fixed using 100% methanol. Then, cells were stained with DAPI for 4 min before being covered with anti-fade fluorescent mounting medium (Dako). Fluorescent signals were visualized by an automated microscope (Biotek, Agilent Technologies, Santa Clara, CA, USA).

### 4.12. siRNA Transfection

Cervical carcinoma cells were transfected with negative control siRNA (Genolution Pharmaceuticals, Seoul, Korea), HIF-1α and BNIP-3 siRNA using G-Fectin (Genolution) according to the manufacturer’s protocol. After 24 h, the medium was changed, and cells were treated with 20 μM MAC for 24 h before being used in experiments.

### 4.13. Statistical Analysis

All in vitro data were obtained from at least three separate experiments performed in triplicate. Results were analyzed using Graph Pad Prism 5 software (Graph Pad Software, La Jolla, CA, USA). The one-way ANOVA test was used to determine the significance of differences between experimental and control values. * *p* < 0.05 indicates a statistically significant difference between experimental and control values.

## Data Availability

All data included in this study are available from the corresponding author.

## Abbreviations

MAC	4-O-methylascochlorin
Akt	Serine-threonine kinase B
mTOR	mammalian target of rapamycin
p70S6K	70-kDa ribosomal protein S6 Kinase
BNIP-3	Bcl-2/adenovirus E1B 19 kDa protein-interacting protein 3
HIF-1α	Hypoxia-inducible factor-1 alpha
PARP	Poly (ADP-ribose) polymerase
CQ	Chloroquine
ATG	Autophagic-related gene
AMPK	AMP-activated protein kinase
IC_50_	Half-maximal-inhibitory concentrations
LAMP1	Lysosomal-associated membrane protein 1
TFEB	Transcription factor EB
PI	Propidium iodide
DMEM	Dulbecco’s Modified Eagle Medium
RPMI	Roswell Park Memorial Institute
FBS	Fetal bovine serum
PBS	Phosphate-buffered saline
FITC	Fluorescein isothiocyanate
Trypsin EDTA	Trypsin disodium ethylenediaminetetraacetic acid
